# Management of Esophageal Cancer-Associated Respiratory–Digestive Tract Fistulas

**DOI:** 10.3390/cancers14051220

**Published:** 2022-02-26

**Authors:** Julia K. Grass, Natalie Küsters, Fabien L. von Döhren, Nathaniel Melling, Tarik Ghadban, Thomas Rösch, Marcel Simon, Jakob R. Izbicki, Alexandra König, Matthias Reeh

**Affiliations:** 1 Department of General, Visceral and Thoracic Surgery, University Medical Center Hamburg-Eppendorf, Martinistraße 52, 20246 Hamburg, Germany; n.kuesters@uke.de (N.K.); fabien.vondoehren@gmx.de (F.L.v.D.); n.melling@uke.de (N.M.); t.ghadban@uke.de (T.G.); izbicki@uke.de (J.R.I.); alexandra.koenig@klinikum-whv.de (A.K.); m.reeh@uke.de (M.R.); 2 Department of Interdisciplinary Endoscopy, University Medical Center Hamburg-Eppendorf, Martinistraße 52, 20246 Hamburg, Germany; t.roesch@uke.de; 3 Department of Respiratory Medicine, University Medical Center Hamburg-Eppendorf, Martinistraße 52, 20246 Hamburg, Germany; m.simon@uke.de

**Keywords:** esophageal cancer, respiratory–digestive tract fistula, tracheoesophageal fistula, tracheobronchial fistula, esophagorespiratory fistula, management strategies

## Abstract

**Simple Summary:**

As rare but life-threatening complications, respiratory–digestive tract fistulas (RDF) have a major impact on esophageal cancer patients. Furthermore, interdisciplinary treatment concepts are still evolving. This retrospective study aims to assess general strategies for RDF, especially in terms of technical and anatomical approaches. In 51 RDF patients, we proved that bilateral fistula repair and combined surgical and non-surgical intervention correlated significantly with good short- and long-term outcomes.

**Abstract:**

Respiratory–digestive tract fistulas are fatal complications that occur in esophageal cancer treatment. Interdisciplinary treatment strategies are still evolving, especially in anatomical treatment stratification. Thus, this study aims to evaluate general therapeutic strategies for this rare condition. Medical records were reviewed for esophageal cancer-associated respiratory–digestive tract fistula patients treated between January 2008 and September 2021. Fistulas were classified according to being surgery- and tumor-associated. Treatment strategies, clinical success, and survival were analyzed. A total of 51 patients were identified: 28 had tumor-associated fistulas and 23 surgery-associated fistulas. Risk factors for fistula development such as radiation (OR = 0.290, p = 0.64) or stent implantation (OR = 1.917, *p* = 0.84) did not correlate with lack of symptom control for RDF patients. In contrast, advanced lymph node metastasis as another risk factor was associated with persistent symptoms after treatment for RDF patients (OR = 0.611, *p* = 0.01). Clinical success significantly correlated with bilateral fistula repair in surgery-associated fistulas (*p* = 0.01), while tumor-associated fistulas benefited the most from non-surgical (*p* = 0.04) or combined surgical and non-surgical intervention (*p* = 0.04) and a bilateral fistula repair (*p* = 0.02) in terms of overall survival. The therapeutic strategy should aim for bilateral fistula closure. A multidisciplinary, stepwise approach might have the best chance for restoration or symptom control with optimized overall survival in selected patients.

## 1. Introduction

Respiratory–digestive tract fistulas (RDF) are among the most fatal and life-threatening complications during the treatment of esophageal carcinoma (EC). The pathological communication between the respiratory and digestive tract affects 5–22% of patients with advanced EC [1,2,3,4,5,6], potentially underestimating the de facto incidence as autopsy data have shown [6]. Risk factors are advanced tumor and nodal status [7,8,9], esophageal stenosis causing friction during food passage [7,9,10], deep tumor ulcerations [10,11], adjuvant or definitive radiochemotherapy [6,8,9,10,12,13] and previous esophageal stent placement [14,15]. Following esophagectomy, 0.3–3.9% [16,17,18,19,20] of patients develop RDF as a consequence of tracheobronchial devascularization, anastomotic leakage, and gastric conduit necrosis driven by mediastinitis and inflammatory involvement of the tracheobronchial system [17,20]. Typical symptoms are severe cough after food intake, frequent aspiration, and purulent pneumonia [1,21,22]. Furthermore, rapidly progressive septic diseases might also result from continuous contamination of the respiratory tract, mediastinum, and pleural cavity. RDF summarizes a morbid condition, which is a negative predictor for long-term outcome. After tumor-associated RDF (T-RDF) diagnosis, survival with supportive therapy alone is reported to range between 1 and 8 weeks [1,5,22,23,24,25,26,27], which can be extended with non-surgical treatment, if clinically successful, up to 3.4–7.9 months [21,26,28,29]. In well-selected patients, surgical intervention has resulted in significantly longer survival of 10–105.0 months [15,30,31]. For surgery-associated RDF (S-RDF), overall mortality has been reported to range between 25.0 and 57.1%, with inconsistent results regarding surgical (17.4–100.0%) and non-surgical (25.0–28.3%) outcome [18,32,33].

A wide range of therapeutic approaches has been described for RDF, from fibrin glue application over stenting to esophageal diversion. However, general treatment strategies have not been described so far. In particular, little is known about anatomical treatment stratification, whether only one or both fistula orifices should be sealed. Therefore, this study is the first to evaluate the general multidisciplinary treatment principles of respiratory–digestive tract fistulas in esophageal cancer patients.

## 2. Materials and Methods

All patients treated for esophageal cancer-associated respiratory–digestive fistula at the University Medical Center Hamburg-Eppendorf between January 2008 and September 2021 were identified from surgical, endoscopic, radiological, and hospital databases. Patients with benign acquired and congenital fistulas were excluded. The medical records at our institution were reviewed entirely for each included patient.

According to local laws, no informed patient consent or statement by the federal ethics committee is required since the study is non-interventional and retrospective (§12HmbKHG—city law Hamburg).

Studied variables included gender, age, date of birth, death, and date of the last follow-up, coexisting medical conditions by Charlson Comorbidity Index (CCI) calculated at fistula diagnosis, tumor stage administered by the American Joint Committee on Cancer Union 6th–8th edition depending on the year of diagnosis, tumor localization, history of definitive chemoradiation or multimodal therapy and laboratory results.

Fistulas were subdivided into T-RDF and S-RDF. T-RDF also included patients who developed fistulas after esophagectomy due to local recurrence without evidence of leakage or conduit necrosis. Tracheobronchial localization according to Wang et al. [21] and fistula diameter were recorded. Patients’ pretherapeutic condition and therapeutic approaches, along with the time interval between diagnostical and therapeutic steps, were analyzed. The curative status of the underlying disease was defined as a locally resectable tumor and the absence of distant metastases. Non-surgical techniques were defined as endoscopic or bronchoscopic interventions. Furthermore, the anatomical treatment approach was evaluated and classified in treatments sealing only the gastrointestinal orifice, those sealing only the respiratory orifice or bilateral approaches and conservative treatment with the best supportive care. The treatment strategy in cases of local recurrence included chemoradiation, surgical reintervention, and best supportive care depending on previous therapy, patient condition, and morbidity caused by the RDF.

Outcomes were analyzed as ‘restoration or symptom control’, ‘no symptom control’, and ‘30-d mortality’ after treatment. Symptom control was defined as the capability of oral food intake and absence of respiratory infection after the resumption of oral cost. For subgroup analysis of T-RDF, only symptom control was evaluated since the underlying disease of advanced EC limited survival. The therapeutic outcome was investigated by occurrence and severity of complications according to Clavien-Dindo classification and 30-day mortality. Survival analysis was calculated following the date of fistula diagnosis. Anastomotic leakage was diagnosed endoscopically.

Data management and statistical analysis were performed using IBM SPSS Statistics for Macintosh, Version 25.0. (Armonk, NY, USA: IBM Corp.). Descriptive statistics are reported as absolute numbers and percentages or mean and standard deviation (SD), as indicated. For univariate analyses, the ANOVA was applied for the comparison of three groups. For two groups, the Student t test was applied for parametric continuous variables and the Mann–Whitney U test for nonparametric continuous variables. Categorical variables were tested using the *χ^2^* test or the Fisher exact test as appropriate. Survival rates were estimated using the log-rank test and described by Kaplan–Meier curves. A two-sided *p*-value < 0.05 was considered as significant. The study conformed to the standards of the Declaration of Helsinki.

## 3. Results

A total of 51 patients with esophageal cancer-associated RDF were able to be identified: 28 with T-RDF and 23 S-RDF (Table 1). S-RDF had a higher share of lower tumors and esophagobronchial (EBF) rather than esophagotracheal fistula (ETF). Four T-RDF patients (14.3%) had received curative cancer treatment at fistula diagnosis. Moreover, the S-RDF cohort had a significantly poorer condition at diagnosis and a shorter interval from fistula diagnosis to first intervention. Thirty-day mortality was 17.9% in the T-RDF and 30.4% in the S-RDF cohort.

The clinicopathological parameters of the T-RDF cohort are shown in Table 2. Neither patients’ demographics nor tumor parameters correlated with the clinical outcome. Fistula characteristics and patients’ conditions at RDF diagnosis (*p* = 0.443) had no impact on the clinical course after fistula development. In contrast, patients with a curative treatment at fistula diagnosis had a higher chance for cure or symptom control (*p* = 0.016).

For S-RDF, demographics and tumor-associated parameters as the histological subtype (*p* = 0.30), infiltration depth (*p* = 0.51), or lymph node metastasis (*p* = 0.21) also showed no correlation with the outcome (Table 3). All S-RDF were associated with anastomotic leakage or conduit necrosis presenting with either acute (39.9%) or delayed (>30d, 60.9%) onset, which did not correlate with the clinical outcome (*p* = 0.886). However, neoadjuvant radiation as a risk factor for RDF development (*p* = 0.03), advanced UICC stage (*p* = 0.03), and anatomical aspects such as cervical anastomosis (*p* = 0.046) or a higher fistula location (*p* = 0.02) correlated significantly with an unfavorable clinical course. The advanced tumor and nodal stage did not reveal a correlation with poor outcomes. However, all pN2 staged patients died in the postoperative course, while 75.0% of cured patients had no lymph node metastasis (*p* = 0.20). A total of 63.6% of the patients deceased within 30 days presented with severe illness necessitating intensive care treatment at diagnosis of RDF (*p* = 0.02).

The therapeutic strategy was significantly associated with the clinical outcome for both T-RDF and S-RDF.

The lowest therapeutic success rates were found in T-RDF patients treated conservatively (0.0%) and solely surgically (16.1%, Table 4). The initial treatment did not correlate with the final outcome (Technique *p* = 0.51, Anatomical treatment *p* = 0.22). In total, 82.1% of T-RDF patients were evaluated for reintervention due to insufficient symptom control, which resulted in 43.5% in reintervention. Though, after the primary intervention, four patients experienced initial symptom control, of which one was of long duration.

Among patients who experienced an overall symptom control, surgical reinterventions (80.0%, *p* < 0.01) and bilaterally fistula repair (80.0%, *p* = 0.03) were the most frequent strategies. Though, both were accompanied by a high peri=interventional risk. Change in therapeutic strategy in cases of unfavorable results led to 50.0% restoration or symptom control and showed a trend towards favorable outcome (*p* = 0.05). For T-RDF, no non-surgical technique correlated with restoration or symptom control. Suture of the esophageal (*p* < 0.01) or respiratory fistula orifice (*p* = 0.04) and the interposition of latissimus dorsi (*p* < 0.01) were associated with a favorable outcome. Moreover, esophageal diversion was also associated with a good outcome (*p* = 0.04).

Sealing both the esophageal and respiratory fistula orifice was by far the most frequently applied anatomical approach among cured or symptom-controlled S-RDF patients as an initial (87.5%, *p* = 0.046) or step-up approach (100.0%, *p* = 0.01, Table 5). On the other hand, 50.0% of bilaterally treated patients died in the postoperative course (30-d mortality). S-RDF patients benefited from bilateral fistula repair both in the first treatment attempt (*p* = 0.046) and overall (*p* = 0.01, Table 5). The choice of intervention had no impact on the final outcome in this cohort (*p* = 0.57, *p* = 0.09, and *p* = 0.11). S-RDF benefited, whenever possible, from esophageal segment resection and re-anastomosis (*p* = 0.04). An esophageal diversion was applied as an ultima ratio in eight patients, five (62.5%) of whom nevertheless died. One of the surviving patients could be reconstructed successfully. Moreover, soft tissue flaps were also associated with restoration or symptom control (*p* = 0.01).

Complications and their severity are presented in Tables S1 and S2. Patients with no options for therapeutic interventions were at the highest risk for mortality (60.0%). However, surgery was accompanied by an elevated risk of peri-interventional mortality compared to non-surgical intervention (3.3% vs. 26.7%). Of note, overall severe complications were more frequent following non-surgical intervention (*p* = 0.02). After the final treatment, no significant difference between anatomical approaches (*p* = 0.11) or techniques (*p* = 0.16) was found.

**Figure 1:**
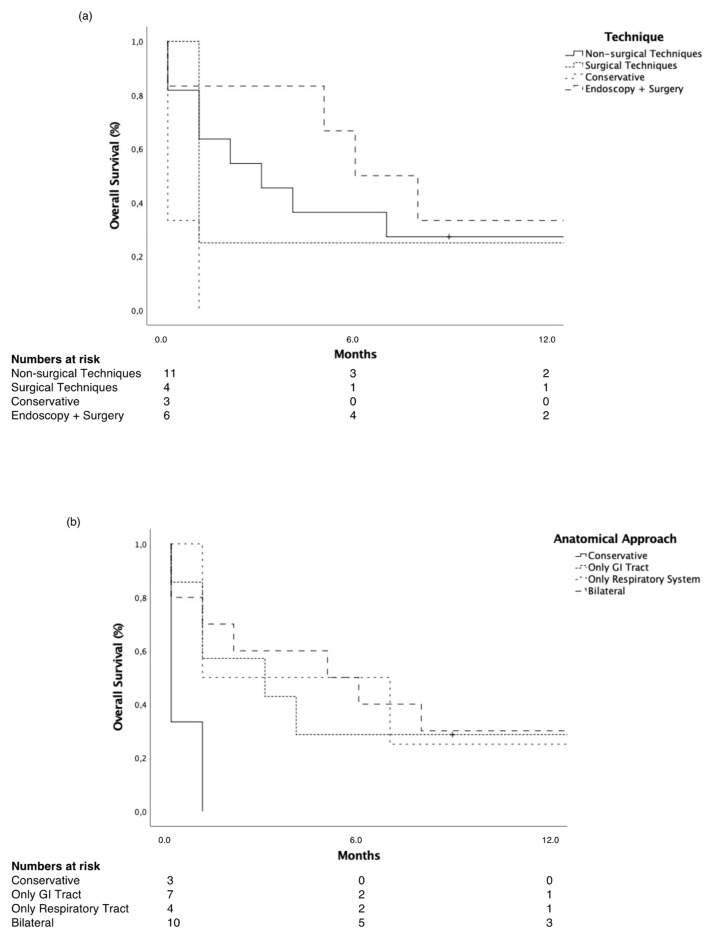
Overall survival by (**a**) interventional technique and (**b**) anatomical approach in T-RDF. (**a**) Non-surgical (*p* = 0.04) and combined endoscopic and surgical intervention (*p* = 0.04) proved significantly longer survival compared to best supportive care. (**b**) Bilateral fistula repair was associated with significantly longer survival compared to best supportive care (*p* = 0.02). GI—gastrointestinal; T-RDF—tumor-associated respiratory–digestive tract fistula.

For overall survival, only a bilateral fistula repair compared to conservative treatment reached statistical significance in T-RDF patients (*p* = 0.02, Figure 1). Combined surgical and non-surgical techniques were significantly associated with long-term survival (*p* = 0.04) and endoscopic intervention (*p* = 0.04), both compared to best supportive care.

**Figure 2:**
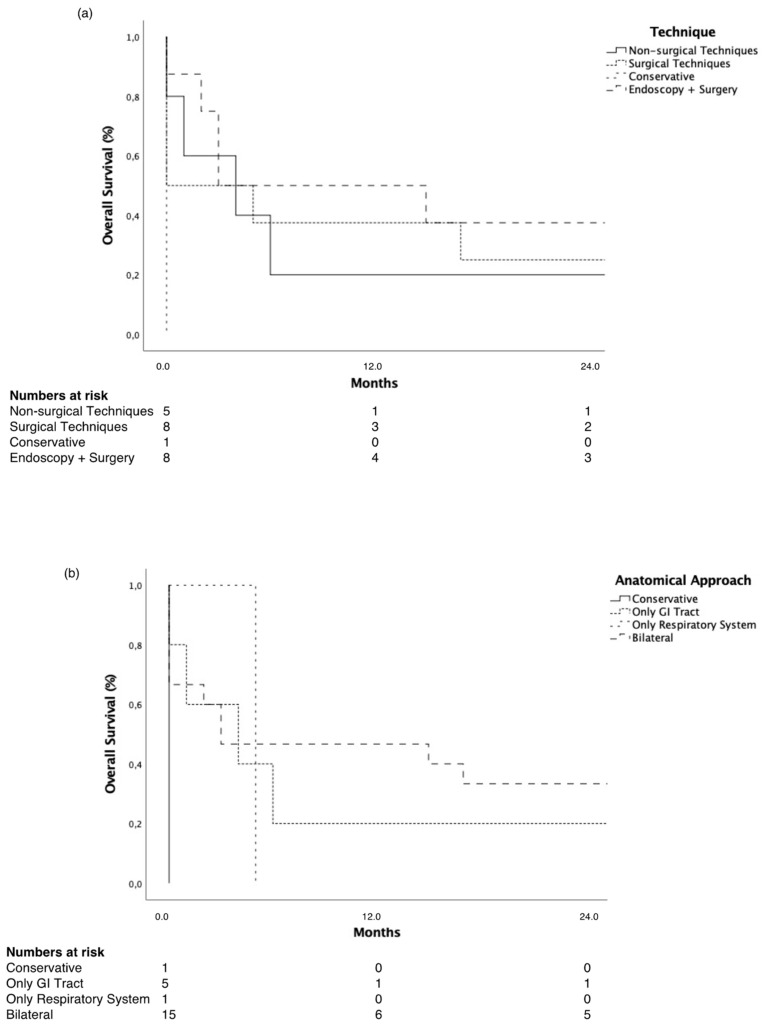
Overall survival by (**a**) interventional technique and (**b**) anatomical approach in S-RDF. (**a**) All interventional techniques were associated with significantly longer overall survival compared to best supportive care (non-surgical techniques *p* = 0.03, surgical techniques *p* = 0.005, combined endoscopic and surgical techniques *p* = 0.005). (**b**) Bilateral (*p* < 0.001) and only GI tract approaches (*p* = 0.03) were significantly associated with improved survival compared to best supportive care. GI—gastrointestinal; S-RDF—surgery-associated respiratory–digestive tract fistula.

In the S-RDF cohort, all techniques proved to have significantly longer survival compared to best supportive care. Moreover, no differences could be found between the interventional techniques (Figure 2). Moreover, bilateral anatomical approaches (*p* < 0.01) and the sole sealing of the gastrointestinal fistula orifice (*p* = 0.03) induced significantly better survival rates compared to best supportive care.

## 4. Discussion

Respiratory–digestive tract fistulas are associated with high morbidity and mortality. Treatment of this fragile condition often fails to reach persistent symptom control, and approaches for strategic interdisciplinary therapy are currently still lacking. This study demonstrates that as far as the patient’s condition allows, a bilateral fistula repair is associated with optimized outcomes for most RDF patients.

Several studies already investigated procedural safety, success rates or risk factors of specific treatment options in case series [3,4,5,6,7,16,21,23,26,27,29,30,31]. However, especially for T-RDF, comparative data of multidisciplinary treatments for short- and long-term outcomes are missing. Moreover, no previous study fully addressed anatomical considerations of RDF therapy: whether one or both fistula orifices should be sealed. This has been underlined by several studies, which concluded that the appropriate management of RDF is still uncertain and variable [18,32,34].

With best supportive care alone, the mean survival of T-RDF was 25.7 ± 13.5 days, which is in line with previous reports [5,23,26]. Non-surgical and surgical therapy can prolong the mean survival to 11.7 ± 6.6 (*p* = 0.04) and 19.0 ± 15.6 months. Previous publications could reveal 2.3–7.9 months after interventional treatment [15,21,26,28] and up to 10 months median survival after surgery [15,30,31]. Moreover, combined surgical and non-surgical therapy could extend the mean survival to 27.8 ± 13.4 months (*p* = 0.04), which has not been demonstrated so far. Similarly, a bilateral fistula repair resulted in significantly prolonged survival (24.4 ± 10.3, *p* = 0.03). In patients with S-RDF, long-term outcome was significantly improved following bilateral fistula repair (*p* < 0.001) or sole sealing of the gastrointestinal fistula orifice (*p* = 0.03). All interventional techniques could prove significantly longer survival compared to best supportive care. A previous study aligns with this finding [15], while a meta-analysis of 89 S-RDF patients demonstrated longer survival after surgery compared to bronchial stenting [32].

However, symptom control is the best prognostic factor for long-term survival [29]. The key principle of the RDF treatment strategies must be airway protection to reach patient stabilization and reduce infectious complications [33,34].

Only a small number of interventions were successful in the first attempt, and in 37.5% of these cases, a reintervention was necessary for recurrent symptoms. Conversion from interventional to surgical therapy or vice versa had a beneficial impact on the clinical success rate in the T-RDF cohort, suggesting that the stepwise approach should be considered the pivotal therapeutic concept for these patients. In most converted cases, patients who were not primarily surgical candidates profited from a reduction in tracheobronchial contamination by esophageal stenting and were later amenable for surgical fistula repair. For S-RDF, therapy conversion resulted in 50.0% therapeutic success.

The literature emphasizes the value of esophageal stenting for RDF therapy: initial symptom control rates of up to 90% have been published [3,5,6,18,23,26,29], although fistula recurrence is frequent six weeks after stent placement [26,33]. Our data align with these results: 68% of stents were not associated with symptom control, but esophageal stent placement improved mean survival to 11.9 ± 6.5 months in T-RDF patients, which corresponds approximately to the 1.5–5-fold survival in the literature [15,26,28,29]. On the other hand, in 50% of symptom-controlled patients, esophageal stents were part of a multidisciplinary approach, which extended mean survival up to 28.2 ± 13.3 months.

Soft tissue flaps have been discussed as an effective treatment for RDF or as fistula prevention in high-risk esophagectomies [19,35]. Sealing both fistula orifices at once was the only procedure significantly associated with the clinical success rate (71.4%). Nevertheless, the failure of soft tissue flaps is high: 30-d mortality rate is 50.0% in S-RDF and 20.0% in T-RDF, and 21.1% and 40.0%, respectively, lack symptom control. Thus, no single technique can be promoted for RDF therapy, but soft tissue flaps can serve as an effective procedure in a multidisciplinary approach.

Peri-interventional complications were frequent in this frail cohort: the share of complication-free and primarily successful interventions was highest among surgical (33.3%) and bilateral procedures (25.7%). While the severity of complications did not differ widely between surgical and non-surgical techniques, high rates of overall peri-interventional mortality accompany both surgical (31.0%) and bilateral fistula repair (31.4%). Nonetheless, these numbers compete with a 60% 30-d mortality for primary best-supportive care.

All S-RDF cases were associated with acute or chronic anastomotic leakage, which might predispose inflammatory affection of the mediastinum and the tracheobronchial system and may account for the poorer general condition of S-RDF compared to T-RDF patients. Consequently, therapy indication and initiation were faster (0.6 ± 1.3 vs. 27.7 ± 51.5 days) in S-RDF patients, which nonetheless were at the highest risk for 30-d mortality (87.5%, *p* = 0.02). Rapid therapeutic onset is inversely correlated with the outcome of S-RDF (*p* = 0.02), which instead reflects the critical condition forcing an immediate therapy than should be attributed to the quick intervention itself. This underlines the value of an early RDF diagnosis and therapy initiation as the most effective steps for patient survival. Once RDF leads to decompensation, patients who are not amenable to radical treatment strategies or therapies are less likely to succeed. The pulmonary function often presents a limiting factor, omitting single-lung ventilation or apnea phases for bronchoscopic or surgical sealing of the respiratory orifice.

Risk factors for T-RDF development, such as radiation [6,8,9,10,12,13] (*p* = 0.63), previous esophageal stent placement [14,23] (*p* = 0.84), squamous cell cancer (SCC) as histological subtype [36] (*p* = 0.29) or T-stage [7,8,9] (*p* = 0.43) did not have an impact on the clinical outcome. In contrast, an advanced lymph node status [9] (*p* = 0.05) came close to statical significance, which has previously been associated with an extended radiation field [9]. In fact, seven of eight patients with advanced lymph node metastasis received radiation before fistula development.

For S-RDF patients, neoadjuvant radiation as a risk factor for fistula development [16,17,20] significantly correlated with poor outcomes (*p* = 0.03). Only a sizeable total tumor burden significantly correlated from several factors associated with compromised perfusion of the tracheobronchial system, such as extended lymphadenectomy or dissection, advanced lymph node metastasis, or large tumors [17,20] with poor clinical outcome (UICC, *p* = 0.03). This underlines the negative impact of surgical devascularization of the tracheobronchial system on the healing capacity. While fistulas originating from cervical anastomoses and those leading into the trachea were at higher risk for lack of symptom control, 30-d mortality was lower than thoracical anastomoses and EBF. Limited therapeutic and endoscopic options might explain this in cases with cervical anastomosis [21,26] and a lower transition of digestive fluids in descending compared to horizontal fistulas [33].

The presented study has several limitations. First, the retrospective character and the cohort size restrain this research, which hampers the correct assessment of clinical success. Clinical follow-up was not utterly accessible in some patients, either due to symptom control or avoidance of further medical contact or practitioner change. Moreover, one patient without symptom control in the clinical follow-up survived 60.6 months, so a secondary restoration can be assumed but not proven.

## 5. Conclusions

Overall, this study is the first to analyze general treatment stratification and multidisciplinary approaches for RDF therapy. Sufficient fistula repair was achieved most effectively by bilateral surgical procedures in both cohorts. However, patient condition and palliative disease at diagnosis limit the therapeutic success. Radical approaches should be reserved for selected patients but can be offered to a broader range of patients in a stepwise, multidisciplinary approach. With a bilateral fistula repair, the optimized long-term outcome can be achieved for both tumor- and surgery-associated fistulas.

## Figures and Tables

**Table 1:** Demographic, clinicopathological, and RDF parameters.

Parameter	T-RDF	S-RDF	*p*-Value
	*n* = 28	*n* = 23	
Age (y)	60.6 ± 7.3	62.9 ± 10.4	0.41
Gender			
*female*	9 (32.1)	5 (21.7)	0.53
*male*	19 (67.9)	18 (78.3)	
CCI			
*0–2*	14 (50.0)	14 (60.9)	0.57
*≥3*	14 (50.0)	9 (39.1)	
Histology			
*AC*	5 (17.9)	13 (56.5)	**0.009**
*SCC*	20 (71.4)	10 (43.5)	
*missing data*	3 (10.7)	0 (0.0)	
UICC			
*I*	0 (0.0)	8 (34.8)	**<0.001**
*II*	4 (14.3)	7 (30.4)	
*III*	5 (17.9)	6 (26.1)	
*IV*	17 (60.7)	1 (4.3)	
*data missing*	2 (7.1)	1 (4.3)	
Tumor localization			
*upper third*	6 (21.4)	2 (8.7)	**0.011**
*middle third*	12 (42.9)	4 (17.4)	
*lower third*	8 (28.6)	17 (73.9)	
RDF			
*ETF (I–IV)*	18 (64.3)	5 (21.7)	**0.004**
*EBF (V–VIII)*	9 (32.1)	18 (78.3)	
*combined*	1 (3.6)	0 (0.0)	
RDF size at diagnosis (mm)	9.7 ± 9.5	6.9 ± 8.3	0.14
Palliative disease at RDF diagnosis			
*no*	4 (14.3)	21 (91.3)	**<0.001**
*yes, for malignant disease*	23 (82.1)	1 (4.3)	
*yes, unfit for therapy*	1 (3.6)	1 (4.3)	
ΔEC diagnosis to RDF diagnosis (d)	442.6 ± 581.4	253.1 ± 565.9	**0.03**
Patient condition at diagnosis			
*normal ward, no signs for relevant infection*	10 (35.7)	6 (26.1)	**0.03**
*normal ward, infection signs ^#^*	16 (57.1)	7 (30.4)	
*ICU surveillance*	0 (0.0)	2 (8.7)	
*ICU therapy*	0 (0.0)	4 (17.4)	
*shock, severe organ dysfunction ^§^*	2 (7.1)	4 (17.4)	
CRP at diagnosis (mg/L)	107.6 ± 98.3	131.6 ± 108.1	0.48
ΔRDF diagnosis to primary treatment (d)	27.7 ± 51.5	0.6 ± 1.3	**0.001**
Patient condition at primary treatment			
*no ICU treatment*	26 (92.9)	15 (65.2)	**0.013**
*ICU treatment, mech. ventilation*	2 (7.1)	8 (34.8)	
CRP at primary treatment (mg/L)	107.5 ± 113.8	132.2 ± 107.3	0.21
ΔRDF diagnosis to end of therapy (d)	91.2 ± 80.0	117.5 ± 204.6	0.58
30-d mortality	5 (17.9)	7 (30.4)	0.34

Numbers are presented as mean ± standard deviation or absolute numbers and percentages in paracenteses. *p*-values in bold indicate statistical significance between cohorts. AC—adenocarcinoma; CCI—Charlson Comorbidity Index; EBF—esophagobronchial fistula; ETF—esophagotracheal fistula; d—days; EC—esophageal cancer; ICU—intensive care unit; l—liter; mg—milligram; mm—millimeter; T-RDF—tumor-associated respiratory–digestive tract fistula; *n*—patient number; S-RDF—surgery-associated respiratory–digestive tract fistula; SCC—squamous cell cancer; UICC—Union internationale contre le cancer, y—years. # (fever, CRP > 50 mg/dL) or any infection-associated organ dysfunction; § (FiO2 > 0.5, noradrenaline > 20 µg/min).

**Table 2:** Correlation of demographic, clinicopathological, and RDF parameters, and risk factors with clinical outcome in T-RDF patients.

Parameter	Restoration or Symptom Control	No Symptom Control	*p*
	*n* = 6	*n* = 22	
Age (y)	57.2 ± 7.6	61.5 ± 7.1	0.20
Gender			
*female*	2 (33.3)	7 (31.8)	0.94
*male*	4 (66.7)	15 (68.2)	
CCI			
*0–2*	4 (66.7)	10 (45.5)	0.36
*≥3*	2 (33.3)	12 (54.5)	
Tumor localization			
*upper third*	2 (33.3)	4 (18.2)	0.25
*middle third*	1 (16.7)	11 (50.0)	
*lower third*	3 (50.0)	5 (22.7)	
Histology			
*AC*	0 (0.0)	5 (22.7)	0.29
*SCC*	6 (100.0)	14 (63.6)	
Grading			
*G1*	0 (0.0)	0 (0.0)	0.63
*G2*	3 (50.0)	9 (40.9)	
*G3*	3 (50.0)	10 (45.5)	
*no grading after neoadjuvant Tx*	0 (0.0)	3 (13.6)	
T			
*T0*	0 (0.0)	1 (4.5)	0.43
*T1*	0 (0.0)	1 (4.5)	
*T2*	1 (16.7)	0 (0.0)	
*T3*	3 (50.0)	9 (40.9)	
*T4*	2 (33.3)	9 (40.9)	
*missing data*	0 (0.0)	2 (9.1)	
N			
*Nx, N0–N1*	6 (100.0)	11 (50.0)	0.05
*N2–N3*	0 (0.0)	8 (36.4)	
*missing data*	0 (0.0)	3 (13.6)	
M			
*M0*	5 (83.3)	14 (63.6)	0.56
*M1*	1 (16.7)	5 (22.7)	
*missing data*	0 (0.0)	3 (13.6)	
UICC			
*I*	0 (0.0)	0 (0.0)	0.12
*II*	1 (16.7)	3 (13.6)	
*III*	3 (50.0)	2 (9.1)	
*IV*	2 (33.3)	15 (68.2)	
*missing data*	0 (0.0)	2 (9.1)	
Radiotherapy	4 (66.7)	16 (72.7)	0.64
Chemotherapy	3 (50.0)	(0.0)	0.12
Esophageal stent before RDF	3 (50.0)	10 (45.5)	0.84
Non-surgical intervention before RDF	3 (50.0)	14 (63.6)	0.88
Palliative status at RDF diagnosis			
*No*	3 (50.0)	1 (4.5)	**0.02**
*For malignant disease*	3 (50.0)	20 (90.9)	
*Unfit for therapy*	0 (0.0)	1 (4.5)	
∆EC diagnosis to RDF diagnosis (d)	602.4 ± 515.7	402.6 ± 602.2	0.15
∆RDF diagnosis to primary RDF treatment (d)	46.5± 70.0	21.0 ± 44.0	0.31
RDF Type			
*ETF (I–IV)*	4 (66.7)	14 (63.6)	0.87
*EBF (V–VII)*	2 (33.3)	7 (31.8)	
combined	0 (0.0)	1 (4.5)	
RDF size at diagnosis (mm)	7.5 ± 8.0	10.4 ± 10.0	0.32
Patient condition at RDF diagnosis			
*no ICU treatment*	6 (100.0)	20 (90.9)	0.44
*ICU treatment, mechanical Ventilation*	0 (0.0)	2 (9.1)	
CRP at diagnosis (mg/L)	105.5 ± 145.2	108.1 ± 91.0	0.96

Numbers are presented as mean ± standard deviation or absolute numbers and percentages in paracenteses. *p*-values in bold indicate statistical significance between cohorts. AC—adenocarcinoma; CCI—Charlson Comorbidity Index; EBF—esophagobronchial fistula; EC—esophageal cancer; ETF—esophagotracheal fistula; d—days; ICU—intensive care unit; l—liter; mg—milligram; mm—millimeter; T-RDF—tumor-associated respiratory–digestive tract fistula; *n*—patient number; SCC—squamous cell cancer; Tx-therapy UICC—Union internationale contre le cancer, y—years.

**Table 3:** Correlation of demographic, clinicopathological, and RDF parameters and risk factors with clinical outcome in S-RDF patients.

Parameter	Restoration or Symptom Control	No Symptom Control	30-d Mortality	*p*
	*n* = 8	*n* = 4	*n* = 11	
Age (y)	63.8 ± 9.4	64.5 ± 5.1	61.6 ± 12.7	0.87
Gender				
*female*	3 (37.5)	0 (0.0)	2 (18.2)	0.31
*male*	5 (62.5)	4 (100.0)	9 (81.8)	
CCU				
*0–2*	4 (50.0)	3 (75.0)	7 (63.6)	0.68
*≥3*	4 (50.0)	1 (25.0)	4 (36.4)	
Tumor localization				
*upper third*	1 (12.5)	1 (25.0)	0 (0.0)	0.05
*middle third*	3 (37.5)	1 (25.0)	0 (0.0)	
*lower third*	4 (50.0)	2 (50.0)	11 (100.0)	
Histology				
*AC*	3 (37.5)	2 (50.0)	8 (72.7)	0.30
*SCC*	5 (62.5)	2 (50.0)	3 (27.3)	
Grading				
*G1*	1 (12.5)	0 (0.0)	1 (9.1)	0.86
*G2*	3 (37.5)	2 (50.0)	4 (36.4)	
*G3*	3 (37.5)	0 (0.0)	4 (36.4)	
*no grading after neoadjuvant Tx*	1 (12.5)	2 (50.0)	2 (18.2)	
pT				
*pT0*	1 (12.5)	0 (0.0)	1 (9.1)	0.51
*pT1*	4 (50.0)	0 (0.0)	3 (27.3)	
*pT2*	1 (12.5)	2 (50.0)	2 (18.2)	
*pT3*	2 (25.0)	2 (50.0)	5 (45.5)	
pN				
*pN0–pN1*	8 (100.0)	4 (100.0)	7 (63.6)	0.07
*pN2*	0 (0.0)	0 (0.0)	4 (36.4)	
UICC				
*I*	5 (62.5)	0 (0.0)	3 (27.3)	**0.03**
*II*	2 (25.0)	2 (50.0)	3 (27.3)	
*III*	1 (12.5)	0 (0.0)	5 (45.5)	
*IV*	0 (0.0)	1 (25.0)	0 (0.0)	
Level of anastomosis				
*cervical*	0 (0.0)	2 (50.0)	1 (9.1)	**0.046**
*thoracic*	8 (100.0)	2 (50.0)	10 (90.9)	
Neoadjuvant radiation	1 (12.5)	3 (75.0)	4 (36.4)	**0.03**
Neoadjuvant chemotherapy	3 (37.5)	3 (75.0)	0 (0.0)	0.16
∆esophagectomy to RDF diagnosis (d)	521.5 ± 899.0	63.8 ± 48.1	126.8 ± 220.1	0.26
Anastomotic leakage or conduit necrosis				
*Acute onset (<30 d)*	3 (33.3)	2 (22.2)	4 (44.4)	0.89
*Delayed onset (≥30 d)*	5 (35.7)	2 (11.9)	7 (50.0)	
∆RDF diagnosis to primary RDF therapy (d)	2.5 ± 2.1	2.5 ± 2.1	0.2 ± 0.4	**0.001**
RDF type				
*ETF (I–IV)*	1 (12.5)	3 (75.0)	1 (9.1)	**0.02**
*EBF (V–VII)*	7 (87.5)	1 (25.0)	10 (90.9)	
RDF size at diagnosis (mm)	8.3 ± 10.3	14.0 ± 9.0	2.7 ± 2.2	0.05
*data missing*	*1 (12.5)*	*0 (0.0)*	*2 (18.2)*	
Patient condition at RDF diagnosis				
*no ICU treatment*	7 (87.5)	4 (100.0)	4 (36.4)	**0.02**
*ICU treatment, mechanical ventilation*	1 (12.5)	0 (0.0)	7 (63.6)	
CRP at RDF diagnosis (mg/L)	96.4 ± 122.5	54.0 ± 40.8	185.4 ± 90.1	0.05

Numbers are presented as mean ± standard deviation or absolute numbers and percentages in paracenteses. *p*-values in bold indicate statistical significance between cohorts. AC—adenocarcinoma; acute anastomotic leakage < 30d; EBF—esophagobronchial fistula; ETF—esophagotracheal fistula; d—days; ICU—intensive care unit; l—liter; mg—milligram; mm—millimeter; *n*—patient number; S-RDF—surgery-associated respiratory–digestive tract fistula; SCC—squamous cell cancer; Tx—therapy; UICC—Union internationale contre le cancer; y—years.

**Table 4:** Therapeutic management and clinical outcome of T-RDF.

Therapeutic Approach	Restoration or Symptom Control	No Symptom Control	Overall	
	*n* = 6	*n* = 22	*n* = 28	*p*
**Primary treatment**				
Technique				
*Non-surgical*	5 (83.3)	14 (63.6)	19 (67.9)	0.51
*Surgical*	1 (16.7)	4 (18.2)	5 (17.9)	
*Conservative*	0 (0.0)	4 (18.2)	4 (14.3)	
Anatomical approach				
*Only GI Tract*	5 (83.3)	9 (40.9)	14 (50.0)	0.22
*Only respiratory*	1 (16.7)	3 (13.6)	4 (14.3)	
*Bilateral*	0 (0.0)	6 (27.3)	6 (21.4)	
*Conservative*	0 (0.0)	4 (18.2)	4 (14.3)	
**Final treatment**				
Technique				
*Non-surgical ^a^*	1 (20.0)	4 (22.2)	5 (21.7)	**0.005 ^b^**
*Surgical ^a^*	4 (80.0)	2 (11.1)	6 (26.1)	
*Conservative ^a^*	0 (0.0)	12 (66.7)	12 (52.2)	
*Healed after primary intervention*	1 (3.6)	0 (0.0)	1 (3.6)	
*Deceased after primary intervention*	0 (0.0)	4 (18.2)	4 (14.3)	
Anatomical approach				
*Only GI Trac t ^a^*	1 (20.0)	2 (11.1)	3 (13.0)	**0.03 ^b^**
*Bilateral ^a^*	4 (80.0)	4 (22.2)	8 (34.8)	
*Conservative ^a^*	0 (0.0)	12 (66.7)	12 (52.2)	
**Overall treatment**				
Technique				
*Non-surgical*	2 (33.3)	11 (50.0)	13 (46.4)	0.35
*Surgical*	1 (16.7)	3 (13.6)	4 (14.3)	
*Non-surgical + Surgical*	3 (50.0)	4 (18.2)	7 (25.0)	
*Conservative*	0 (0.0)	4 (18.2)	4 (14.3)	
Anatomical approach				
*Only GI Tract*	2 (33.3)	6 (27.3)	8 (28.6)	0.71
*Only respiratory*	1 (16.7)	4 (18.2)	5 (17.9)	
*Bilateral*	3 (50.0)	8 (36.4)	11 (39.3)	
*Conservative*	0 (0.0)	4 (18.2)	4 (14.3)	
Therapy Conversion	3 (50.0)	3 (13.6)	6 (21.4)	0.05
**Therapy**				
Non-surgical				
*Esophageal stent overall*	4 (66.7)	12 (54.5)	16 (57.1)	0.60
*Esophageal stent*	4 (66.7)	10 (45.5)	14 (50.0)	0.65
*Esophageal stent + endosponge*	-	-	-	
*Esophageal stent + surgis plug*	-	-	-	
*Esophageal + bronchial stent*	-	2 (9.1)	2 (7.1)	0.44
*Esophageal fibrin glue + clip*	-	1 (94.5)	1 (3.6)	0.60
*Ovescoclip*	1 (16.7)	-	1 (3.6)	0.05
*Endosponge*	-	-	-	
*Tracheal silicon stent*	-	4 (18.2)	4 (14.3)	0.26
*Esophageal tygon tubus*	-	-	-	
Surgical				
*Esophageal diversion*	2 (33.3)	1 (4.5)	3 (10.7)	**0.043**
*Reconstruction in interval possible*	2 (100.0)	-	2 (50.0)	**0.02**
*Reconstruction successful*	2 (100.0)	-	2 (50.0)	
*Esophageal resection + colonic conduit*	-	2 (9.1)	2 (7.1)	0.44
*Esophageal resection + gastric conduit*	-	2 (9.1)	2 (7.1)	0.44
*Esophageal segment resection*	-	-	-	
*Suture trachea / bronchus*	2 (33.3)	1 (4.5)	3 (10.7)	**0.04**
*Suture esophagus*	4 (66.7)	3 (13.6)	7 (25.0)	**0.008**
*Soft tissue flap*	3 (50.0)	6 (27.3)	9(32.1)	0.29
*Muscle flap*	2 (33.3)	3 (13.6)	5 (17.9)	0.26
*Latissimus dorsi flap*	2 (33.3)	-	2 (7.1)	**0.005**
*Sternocleidomastoideus flap*	-	3 (13.6)	3 (10.7)	0.34
*Non muscle flap*	1 (16.7)	4 (18.2)	5 (17.9)	0.93
*Pericardial flap*	1 (16.7)	3 (13.6)	4 (14.3)	0.85
*V azygos flap*	-	1 (4.5)	1 (3.6)	0.60
*Lung resection with RDF bearing bronchial segment*	-	-	-	

Numbers are presented as mean ± standard deviation or absolute numbers and percentages in paracenteses. *p*-values in bold indicate statistical significance between cohorts. d—days; T-RDF—malignant respiratory–digestive tract fistula; *n* = patient number. ^a^ Percentage of patients with therapeutic re-evaluation without mortality or therapeutic success after first attempt. ^b^ Only re-evaluated patients considered.

**Table 5:** Therapeutic management and clinical outcome of S-RDF.

Therapeutic Approach	Restoration or Symptom Control	No Symptom Control	Death	Overall	*p*
	*n* = 8	*n* = 4	*n* = 11	*n* = 23	
**Primary treatment**					
Technique					
*Non-surgical*	3 (37.5)	3 (75.0)	6 (54.5)	12 (52.2)	0.57
*Surgical*	5 (62.5)	1 (25.0)	4 (36.4)	10 (43.5)	
*Conservative*	0 (0.0)	0 (0.0)	1 (9.1)	1 (4.3)	
Anatomical approach					
*Only GI Tract*	1 (12.5)	3 (75.0)	5 (45.5)	9 (39.1)	**0.046**
*Only Respiratory System*	0 (0.0)	1 (25.0)	0 (0.0)	1 (4.3)	
*Both*	7 (87.5)	0 (0.0)	5 (45.5)	12 (52.2)	
*Conservative*	0 (0.0)	0 (0.0)	1 (9.1)	1 (4.3)	
**Final treatment**					
Technique					
*Non-surgical ^a^*	1 (25.0)	1 (25.0)	1 (14.3)	3 (13.0)	0.09 ^b^
*Surgical ^a^*	3 (75.0)	0 (0.0)	5 (45.5)	8 (34.8)	
*Conservative ^a^*	0 (0.0)	3 (75.0)	1 (14.3)	4 (17.4)	
*Healed after primary intervention*	0 (0.0)	0 (0.0)	0 (0.0)	0 (0.0)	
*Deceased after primary intervention*	4 (50.0)	0 (0.0)	4 (36.4)	8 (34.7)	
Anatomical approach					
*Only GI Tract ^a^*	0 (0.0)	1 (25.0)	1 (14.3)	2 (8.7)	0.36 ^b^
*Bilateral ^a^*	4 (100.0)	0 (0.0)	5 (71.4)	9 (39.1)	
*Conservative ^a^*	0 (0.0)	3 (75.0)	1 (14.3)	4 (17.4)	
**Overall treatment**					
Technique					
*Non-surgical*	0 (0.0)	3 (75.0)	2 (18.2)	5 (21.7)	0.11
*Surgical*	4 (50.0)	1 (25.0)	4 (36.4)	9 (39.1)	
*Non-surgical + Surgical*	4 (50.0)	0 (0.0)	4 (36.4)	8 (34.8)	
*Conservative*	0 (0.0)	0 (0.0)	1 (9.1)	1 (4.3)	
Anatomical approach					
*Only GI Tract*	0 (0.0)	3 (75.0)	2 (18.2)	5 (21.7)	**0.01**
*Only Respiratory System*	0 (0.0)	1 (25.0)	0 (0.0)	1 (4.3)	
*Bilateral*	8 (100.0)	0 (0.0)	8 (72.7)	16 (69.6)	
*Conservative*	0 (0.0)	0 (0.0)	1 (9.1)	1 (4.3)	
Therapy Conversion	4 (50.0)	0 (0.0)	4 (36.4)	8 (34.8)	0.23
**Therapy**					
Non-surgical					
*Esophageal stent overall*	3 (37.5)	2 (50.0)	4 (36.4)	9 (39.1)	0.87
*Esophageal stent*	1 (12.5)	2 (50.0)	3 (27.3)	6 (26.1)	0.38
*Esophageal stent + endosponge*	2 (25.0)	-	-	2 (8.7)	0.13
*Esophageal stent + surgis plug*	-	-	1 (9.1)	1 (4.3)	0.57
*Esophageal + bronchial stent*	-	-	-	-	
*Esophageal fibrin glue + clip*	1 (12.5)	1 (25.0)	1 (9.1)	3 (13)	0.72
*Ovescoclip*	-	-	-	-	
*Endosponge*	-	-	1 (9.1)	1 (4.3)	0.57
*Tracheal silicon stent*	1 (12.5)	-	-	1 (4.3)	0.38
*Esophageal tygon tubus*	-	1 (25.0)	1 (9.1)	2 (8.7)	0.35
Surgical					
*Esophageal diversion*	3 (37.5)	-	5 (45.5)	8 (34.8)	0.26
*Reconstruction in interval possible*	2 (66.7)	-	-	2 (25.0)	0.23
*Reconstruction successful*	1 (33.3)	-	-	1 (12.5)	
*Esophageal resection + colonic conduit*	-	-	1 (9.1)	1 (4.3)	0.57
*Esophageal resection + gastric conduit*	-	-	-	-	
*Esophageal segment resection*	3 (37.5)	-	-	3 (13)	**0.04**
*Suture trachea/bronchus*	2 (25.0)	-	2 (18.2)	4 (17.4)	0.56
*Suture esophagus*	2 (25.0)	-	3 (27.3)	5 (21.7)	0.51
*Soft tissue flap*	7 (87.5)	-	7 (63.6)	14 (60.9)	**0.01**
*Muscle flap*	3 (37.5)	-	4 (36.4)	7 (30.4)	0.35
*Latissimus dorsi flap*	3 (37.5)	-	4 (36.4)	7 (30.4)	0.35
*Sternocleidomastoideus flap*	-	-	-	-	
*Non-muscle flap*	5 (62.5)	-	6 (54.5)	11 (47.8)	0.10
*Pericardial flap*	5 (62.5)	-	5 (45.5)	10 (43.5)	0.12
*V azygos flap*	-	-	1 (9.1)	1 (4.3)	0.57
*Lung resection with RDG bearing bronchial segment*	2 (25.0)	-	3 (27.3)	5 (21.7)	0.51

Numbers are presented as mean ± standard deviation or absolute numbers and percentages in paracenteses. *p*-values in bold indicate statistical significance between cohorts. d—days; S-RDF—surgery-associated respiratory–digestive tract fistula; *n* = patient number. ^a^ Percentage of patients with therapeutic re-evaluation without mortality or therapeutic success after first attempt. ^b^ Only re-evaluated patients considered.

## Data Availability

The data presented in this study are available on request from the corresponding author.

## Supplementary Materials

The following are available online at https://www.mdpi.com/article/10.3390/cancers14051220/s1, Table S1. Adverse events by intervention technique and anatomical approach, Table S2. Severity of adverse event by treatment stratification.
