# Supplementary material for: Management of Esophageal Cancer-Associated Respiratory–Digestive Tract Fistulas
Source: Cancers (Basel). 2022 Feb 26;14(5):1220. doi: 10.3390/cancers14051220 (PMC8909259; doi:10.3390/cancers14051220)
Supplement: Supplementary file 1 [file cancers-14-01220-s001.zip › cancers-1555950-supplementary.pdf]

cancers

MDPI

Supplemental Materials

Management of Esophageal Cancer-Associated Respiratory–Digestive Tract Fistulas

Julia K. Grass [1,*], Natalie Küsters [1], Fabien L. von Döhren [1], Nathaniel Melling [1], Tarik Ghadban[1], Thomas Rösch [2], Marcel Simon [3], Jakob R. Izbicki [1], Alexandra König [1] and Matthias Reeh [1]

Table S1. Adverse events by intervention technique and anatomical approach.

											Procedure specific complications			
		No complication n / N (%)	Pneumonia	Abscess / pleural empyema / mediastinitis	Expansion of RDF	Bronchial occlusion	ARDS	Hemorrhage	Sepsis / shock	30d Mortality	Conduit necrosis	Anastomotic leakage	Anastomotic stenosis	Stent migration
No. of interventions at risk											8	8	8	9
Technique	Non-surgical	4 / 39 (10.3)	11 (28.2)	6 (15.4)	2 (5.1)	2 (5.1)	3 (7.7)	2 (5.1)	3 (7.7)	5 (12.8)				3 (33.3)
	Surgical	10 / 30 (33.3)	1 (3.3)				3 (10.0)	2 (6.7)	8 (26.7)	10 (33.3)	1 (12.5)	2 (25.0)	1 (12.5)	
	conservative	1 / 34 (2.9)	4 (11.7)				1 (2.9)		1 (2.9)	8 (23.5)				
Anatomical approach	Only GI Tract	4 / 28 (14.3)	6 (21.4)	4 (14.3)	2 (7.1)		2 (7.1)	1 (3.6)	2 (7.1)	3 (10.7)				3 (33.3)
	Only Respiratory System	1 / 5 (20.0)	1 (20.0)			1 (20.0)	1 (20.0)							
	Bilateral	9 / 35 (25.7)	5 (14.2)	2 (5.7)			3 (8.6)	3 (8.6)	9 (25.7)	12 (34.3)	1 (12.5)	2 (25.0)	1 (12.5)	
	Conservative	1 / 34 (2.9)	4 (11.8)				1 (2.9)		1 (2.9)	8 (23.5)				

Numbers are presented as absolute numbers of interventions and percentages in paracenteses. ARDS – acute respiratory distress syndrome; d – days; GI – gastrointestinal; n- patients number without complications; N – cohort size.

Table S2. Severity of adverse event by treatment stratification.

	Technique				Anatomical Approach				
	**Non-surgical**	**Surgical**	**Conservative**	**p**	**Only GI Tract**	**Only Respiratory System**	**Bilateral**	**Conservative**	**p**
Primary Treatment	31	15	5		23	5	18	5	
Clavien Dindo									
3a	10 (32.3)	2 (13.3)	0 (0.0)	**0.022**	8 (34.8)	1 (10.0)	3 (16.7)	0 (0.0)	0.051
3b	6 (19.4)	3 (20.0)	0 (0.0)		4 (17.4)	1 (20.0)	4 (22.2)	0 (0.0)	
4	9 (29.0)	2 (13.3)	0 (0.0)		7 (30.4)	1 (20.0)	3 (16.7)	0 (0.0)	
5	1 (3.3)	4 (26.7)	3 (60.0)		0 (0.0)	2 (40.0)	5 (27.8)	3 (60.0)	
Final Treatment [b]	8	14	16		5		17	16	
Clavien Dindo									
3a	1 (12.5)	3 (21.4)	2 (12.5)	0.162	0 (0.0)		4 (23.5)	2 (12.5)	0.107
3b	0 (0.0)	0 (0.0)	0 (0.0)		0 (0.0)		0 (0.0)	0 (0.0)	
4	0 (0.0)	2 (14.3)	1 (6.3)		0 (0.0)		2 (11.8)	1 (6.3)	
5	3 (37.5)	5 (35.7)	4 (25.0)		2 (40.0)		6 (35.3)	4 (25.0)	

Numbers are presented absolute numbers and percentages. p-values in bold indicate statistical significance between cohorts referring to the entire Clavien Dindo scale. [b] excluded from analysis: 5 patients healed after primary intervention, 8 patients dead after primary intervention
